# Supplementary material for: Modeling glioblastoma heterogeneity as a dynamic network of cell states
Source: Mol Syst Biol. 2021 Sep 16;17(9):e10105. doi: 10.15252/msb.202010105 (PMC8444284; doi:10.15252/msb.202010105)
Supplement: Supplementary file 6 — Source Data for Figure 5 [file MSB-17-e10105-s004.zip › Figure5A_sourcedata/GSEA_3017/nosigdb_stateA.GseaPreranked.1622795026485/GAROFANO_IAVARONE_PPR_SUBTYPE.html]

Details for gene set GAROFANO\_IAVARONE\_PPR\_SUBTYPE[GSEA]

|  || Dataset | state53017 |
| Phenotype | NoPhenotypeAvailable |
| Upregulated in class | na\_pos |
| GeneSet | GAROFANO\_IAVARONE\_PPR\_SUBTYPE |
| Enrichment Score (ES) | 0.49750018 |
| Normalized Enrichment Score (NES) | 3.9757075 |
| Nominal p-value | 0.0 |
| FDR q-value | 0.0 |
| FWER p-Value | 0.0 |
Table: GSEA Results Summary

  

Fig 1: Enrichment plot: GAROFANO\_IAVARONE\_PPR\_SUBTYPE      
 Profile of the Running ES Score & Positions of GeneSet Members on the Rank Ordered List

  

| PROBE | GENE SYMBOL | GENE\_TITLE | RANK IN GENE LIST | RANK METRIC SCORE | RUNNING ES | CORE ENRICHMENT || 1 | CDK1 |  |  | 3 | 1.006 | 0.0180 | Yes |
| 2 | MKI67 |  |  | 4 | 0.926 | 0.0377 | Yes |
| 3 | KIF11 |  |  | 6 | 0.870 | 0.0550 | Yes |
| 4 | UBE2C |  |  | 7 | 0.793 | 0.0719 | Yes |
| 5 | MCM10 |  |  | 9 | 0.752 | 0.0868 | Yes |
| 6 | PCDH10 |  |  | 14 | 0.734 | 0.0978 | Yes |
| 7 | KPNA2 |  |  | 20 | 0.667 | 0.1062 | Yes |
| 8 | HIST1H4C |  |  | 21 | 0.659 | 0.1203 | Yes |
| 9 | ASPM |  |  | 27 | 0.639 | 0.1281 | Yes |
| 10 | PIF1 |  |  | 30 | 0.627 | 0.1392 | Yes |
| 11 | DNAJC9 |  |  | 34 | 0.615 | 0.1488 | Yes |
| 12 | NCAPG |  |  | 44 | 0.556 | 0.1503 | Yes |
| 13 | HJURP |  |  | 47 | 0.539 | 0.1595 | Yes |
| 14 | CKS1B |  |  | 50 | 0.530 | 0.1684 | Yes |
| 15 | DBF4 |  |  | 55 | 0.514 | 0.1748 | Yes |
| 16 | SMC3 |  |  | 58 | 0.504 | 0.1832 | Yes |
| 17 | KIF14 |  |  | 59 | 0.503 | 0.1939 | Yes |
| 18 | MASTL |  |  | 60 | 0.499 | 0.2045 | Yes |
| 19 | SLIT1 |  |  | 61 | 0.497 | 0.2151 | Yes |
| 20 | CENPE |  |  | 62 | 0.496 | 0.2257 | Yes |
| 21 | CCNA2 |  |  | 65 | 0.495 | 0.2339 | Yes |
| 22 | TPX2 |  |  | 66 | 0.488 | 0.2443 | Yes |
| 23 | MYBL2 |  |  | 69 | 0.486 | 0.2523 | Yes |
| 24 | HIST1H1B |  |  | 77 | 0.475 | 0.2544 | Yes |
| 25 | UBE2T |  |  | 83 | 0.470 | 0.2586 | Yes |
| 26 | DUT |  |  | 87 | 0.466 | 0.2651 | Yes |
| 27 | PLK1 |  |  | 89 | 0.464 | 0.2738 | Yes |
| 28 | CENPF |  |  | 94 | 0.461 | 0.2790 | Yes |
| 29 | TOP2A |  |  | 95 | 0.457 | 0.2888 | Yes |
| 30 | ZWINT |  |  | 106 | 0.447 | 0.2868 | Yes |
| 31 | AURKB |  |  | 108 | 0.446 | 0.2951 | Yes |
| 32 | SEPHS1 |  |  | 114 | 0.437 | 0.2987 | Yes |
| 33 | CDCA8 |  |  | 115 | 0.437 | 0.3080 | Yes |
| 34 | KIF23 |  |  | 116 | 0.436 | 0.3172 | Yes |
| 35 | NSMCE4A |  |  | 117 | 0.435 | 0.3265 | Yes |
| 36 | MELK |  |  | 123 | 0.427 | 0.3298 | Yes |
| 37 | BUB1B |  |  | 129 | 0.421 | 0.3330 | Yes |
| 38 | HIST2H2AC |  |  | 131 | 0.419 | 0.3408 | Yes |
| 39 | HELLS |  |  | 135 | 0.417 | 0.3462 | Yes |
| 40 | RTKN2 |  |  | 141 | 0.413 | 0.3493 | Yes |
| 41 | GTSE1 |  |  | 146 | 0.411 | 0.3534 | Yes |
| 42 | CCNB2 |  |  | 147 | 0.411 | 0.3622 | Yes |
| 43 | MAD2L1 |  |  | 153 | 0.406 | 0.3651 | Yes |
| 44 | KIFC1 |  |  | 160 | 0.402 | 0.3667 | Yes |
| 45 | ARHGAP11A |  |  | 162 | 0.399 | 0.3740 | Yes |
| 46 | KIF2C |  |  | 165 | 0.397 | 0.3802 | Yes |
| 47 | MCM7 |  |  | 171 | 0.394 | 0.3828 | Yes |
| 48 | NDC80 |  |  | 173 | 0.393 | 0.3901 | Yes |
| 49 | CKLF |  |  | 183 | 0.388 | 0.3880 | Yes |
| 50 | BUB1 |  |  | 188 | 0.384 | 0.3915 | Yes |
| 51 | DCLRE1C |  |  | 190 | 0.381 | 0.3985 | Yes |
| 52 | KIF22 |  |  | 197 | 0.377 | 0.3996 | Yes |
| 53 | SUV39H2 |  |  | 205 | 0.371 | 0.3995 | Yes |
| 54 | PTMA |  |  | 206 | 0.371 | 0.4074 | Yes |
| 55 | TRIP13 |  |  | 209 | 0.370 | 0.4130 | Yes |
| 56 | SMC4 |  |  | 217 | 0.367 | 0.4127 | Yes |
| 57 | SPC25 |  |  | 226 | 0.363 | 0.4112 | Yes |
| 58 | INCENP |  |  | 227 | 0.362 | 0.4189 | Yes |
| 59 | NCAPH |  |  | 232 | 0.359 | 0.4220 | Yes |
| 60 | ZNF704 |  |  | 235 | 0.357 | 0.4273 | Yes |
| 61 | CENPA |  |  | 243 | 0.352 | 0.4267 | Yes |
| 62 | H2AFZ |  |  | 245 | 0.351 | 0.4331 | Yes |
| 63 | FEN1 |  |  | 259 | 0.343 | 0.4254 | Yes |
| 64 | SOX11 |  |  | 261 | 0.342 | 0.4315 | Yes |
| 65 | CENPJ |  |  | 270 | 0.339 | 0.4295 | Yes |
| 66 | CLSPN |  |  | 274 | 0.336 | 0.4332 | Yes |
| 67 | DEPDC1B |  |  | 275 | 0.335 | 0.4404 | Yes |
| 68 | ESCO2 |  |  | 282 | 0.333 | 0.4405 | Yes |
| 69 | MND1 |  |  | 304 | 0.324 | 0.4233 | Yes |
| 70 | BRCA2 |  |  | 308 | 0.323 | 0.4267 | Yes |
| 71 | CENPN |  |  | 310 | 0.323 | 0.4324 | Yes |
| 72 | FANCI |  |  | 319 | 0.319 | 0.4300 | Yes |
| 73 | DNA2 |  |  | 321 | 0.318 | 0.4356 | Yes |
| 74 | TNFAIP8L1 |  |  | 336 | 0.314 | 0.4262 | Yes |
| 75 | RFC3 |  |  | 337 | 0.314 | 0.4329 | Yes |
| 76 | ZNF22 |  |  | 339 | 0.313 | 0.4384 | Yes |
| 77 | CDCA5 |  |  | 341 | 0.312 | 0.4439 | Yes |
| 78 | PRC1 |  |  | 350 | 0.310 | 0.4413 | Yes |
| 79 | ECT2 |  |  | 365 | 0.305 | 0.4317 | Yes |
| 80 | CCNF |  |  | 366 | 0.305 | 0.4381 | Yes |
| 81 | CDCA2 |  |  | 369 | 0.304 | 0.4423 | Yes |
| 82 | BRCA1 |  |  | 373 | 0.302 | 0.4453 | Yes |
| 83 | CKAP2 |  |  | 379 | 0.301 | 0.4459 | Yes |
| 84 | RRM2 |  |  | 383 | 0.300 | 0.4489 | Yes |
| 85 | XRCC2 |  |  | 384 | 0.300 | 0.4552 | Yes |
| 86 | CENPW |  |  | 386 | 0.299 | 0.4604 | Yes |
| 87 | CDC6 |  |  | 391 | 0.297 | 0.4622 | Yes |
| 88 | RACGAP1 |  |  | 399 | 0.293 | 0.4604 | Yes |
| 89 | HMGB1 |  |  | 403 | 0.292 | 0.4631 | Yes |
| 90 | CREB5 |  |  | 407 | 0.291 | 0.4659 | Yes |
| 91 | HMGB2 |  |  | 424 | 0.288 | 0.4536 | Yes |
| 92 | POP7 |  |  | 427 | 0.287 | 0.4574 | Yes |
| 93 | NUSAP1 |  |  | 430 | 0.286 | 0.4612 | Yes |
| 94 | NUF2 |  |  | 434 | 0.285 | 0.4638 | Yes |
| 95 | LMNB1 |  |  | 442 | 0.282 | 0.4617 | Yes |
| 96 | CDC25C |  |  | 443 | 0.282 | 0.4677 | Yes |
| 97 | CDC45 |  |  | 446 | 0.282 | 0.4714 | Yes |
| 98 | ANP32E |  |  | 453 | 0.280 | 0.4705 | Yes |
| 99 | GINS1 |  |  | 462 | 0.279 | 0.4672 | Yes |
| 100 | MNS1 |  |  | 464 | 0.278 | 0.4720 | Yes |
| 101 | ATAD2 |  |  | 470 | 0.275 | 0.4721 | Yes |
| 102 | EZH2 |  |  | 471 | 0.275 | 0.4779 | Yes |
| 103 | SKA1 |  |  | 475 | 0.274 | 0.4803 | Yes |
| 104 | MTG1 |  |  | 478 | 0.274 | 0.4839 | Yes |
| 105 | BLM |  |  | 490 | 0.272 | 0.4770 | Yes |
| 106 | KIF4A |  |  | 504 | 0.269 | 0.4678 | Yes |
| 107 | UBE2S |  |  | 507 | 0.269 | 0.4712 | Yes |
| 108 | PLEKHO1 |  |  | 511 | 0.269 | 0.4735 | Yes |
| 109 | ARC |  |  | 514 | 0.269 | 0.4769 | Yes |
| 110 | HAUS8 |  |  | 518 | 0.267 | 0.4791 | Yes |
| 111 | ZNF90 |  |  | 520 | 0.266 | 0.4836 | Yes |
| 112 | MRTO4 |  |  | 527 | 0.265 | 0.4824 | Yes |
| 113 | RCC1 |  |  | 528 | 0.265 | 0.4880 | Yes |
| 114 | SKA3 |  |  | 531 | 0.264 | 0.4913 | Yes |
| 115 | RANBP1 |  |  | 536 | 0.263 | 0.4923 | Yes |
| 116 | KIF15 |  |  | 538 | 0.263 | 0.4968 | Yes |
| 117 | STIL |  |  | 545 | 0.261 | 0.4954 | Yes |
| 118 | CENPO |  |  | 549 | 0.260 | 0.4975 | Yes |
| 119 | TMPO |  |  | 584 | 0.253 | 0.4638 | No |
Table: GSEA details [plain text format]

  

Fig 2: GAROFANO\_IAVARONE\_PPR\_SUBTYPE: Random ES distribution      
 Gene set null distribution of ES for **GAROFANO\_IAVARONE\_PPR\_SUBTYPE**
